# Factors That Improve Chest Computed Tomography-Defined Sarcopenia Prognosis in Advanced Non-Small Cell Lung Cancer

**DOI:** 10.3389/fonc.2021.754975

**Published:** 2021-10-01

**Authors:** Ming Yang, Lingling Tan, Lingling Xie, Song Hu, Dan Liu, Jing Wang, Weimin Li

**Affiliations:** ^1^ The Center of Gerontology and Geriatrics, West China Hospital, Sichuan University, Chengdu, China; ^2^ Precision Medicine Research Center, West China Hospital, Sichuan University, Chengdu, China; ^3^ National Clinical Research Center for Geriatrics (WCH), West China Hospital, Sichuan University, Chengdu, China; ^4^ West China School of Nursing, West China Hospital, Sichuan University/ Department of Oncology, Shangjin Nanfu Hospital, Sichuan University, Chengdu, China; ^5^ Department of Radiology, Shangjin Nanfu Hospital, Sichuan University, Chengdu, China; ^6^ Population Health Sciences, German Center for Neurodegenerative Diseases (DZNE), Bonn, Germany; ^7^ Department of Respiratory and Critical Care Medicine, West China Hospital, Sichuan University, Chengdu, China; ^8^ Institute of Respiratory Health, Frontiers Science Center for Disease-related Molecular Network, West China Hospital, Sichuan University, Chengdu, China; ^9^ The Research Units of West China, Chinese Academy of Medical Sciences, Chengdu, China

**Keywords:** muscle wasting, muscle depletion, image segmentation, lung cancer, prognosis

## Abstract

**Background:**

Whether muscle strength and physical performance should be components of sarcopenia remains controversial. This study evaluated the skeletal muscle index derived from computed tomography images at the 12th thoracic vertebra level (T12 SMI), handgrip strength, performance status, and their combination for predicting overall survival in patients with advanced non-small cell lung cancer.

**Methods:**

Chest computed tomography, handgrip strength measurement, and bioelectrical impedance analysis were performed. Sarcopenia was defined based on the T12 SMI alone or the T12 SMI, handgrip, and/or physical performance (i.e. Asian Working Group for Sarcopenia [AWGS]-defined sarcopenia or severe sarcopenia).

**Results:**

Overall, 639 participants were included; 488 (76.4%) died. At baseline, 160 (25.0%), 141 (22.1%), and 42 (6.6%) patients had computed tomography-defined sarcopenia, AWGS-defined sarcopenia, and AWGS-defined severe sarcopenia, respectively. Chest computed tomography-defined sarcopenia (hazard ratio [HR], 2.00; 95% confidence interval [CI], 1.65-2.43), AWGS-defined sarcopenia (HR, 2.00; 95% CI, 1.59-2.49), and AWGS-defined severe sarcopenia (HR, 3.01; 95% CI, 2.21-4.09) were more strongly associated with poor prognosis than a performance status score ≥2 (HR, 1.37; 95% CI, 1.10-1.73).

**Conclusions:**

Adding handgrip strength and the performance status score to chest computed tomography-defined sarcopenia improved its prognostic ability. Oncological sarcopenia research should focus on muscle mass, strength, and function.

## Introduction

Sarcopenia is a skeletal muscle disorder characterized by a progressive and generalized loss of muscle quantity and quality (1). Sarcopenia is currently regarded as the hallmark of cancer cachexia (2) and has been confirmed to be a prognostic factor for poor outcomes in numerous malignancies (3–5). Although sarcopenia has become a major factor in the fields of oncological and geriatric research, several fundamental questions regarding sarcopenia remain unanswered (1).

The first and foremost question concerns the definition of sarcopenia (6). There currently exist two major opinions about the definition of sarcopenia (7). In the fields of oncology and surgery, most researchers use the term ‘sarcopenia’ to refer to low muscle mass without any measurement of muscle strength or function (7, 8). For instance, the recently published North American Expert Opinion Statement on Sarcopenia in Liver Transplantation recommends defining sarcopenia ‘using only muscle mass’ (9). Conversely, in the fields of geriatric and internal medicine, there is a consensus that sarcopenia should be defined based on low muscle mass, low muscle strength, and/or low physical performance (10, 11). Consequently, this knowledge gap regarding the definition of sarcopenia has hindered interdisciplinary cooperation (7).

Computed tomography (CT) represents one of the gold standard methods for skeletal muscle mass (SMM) measurement (12). The skeletal muscle area (SMA) on a single-slice CT image at the third lumbar vertebra (L3) level is the most widely used indicator in the literature (13) and is conventionally regarded as the *de facto* gold standard in CT body composition assessment (7, 12). Nevertheless, in clinical practice, abdominopelvic CT scans are less frequently performed than chest CT scans, particularly in patients with lung cancer. Furthermore, several studies on lung cancer had to exclude up to one-third of patients because of missing data from CT images at the L3 level (14, 15). Therefore, the identification of an SMM marker based on chest CT in patients with lung cancer would certainly benefit sarcopenia research (16, 17). SMA at the 12^th^ thoracic vertebra level (T12 SMA) has recently been shown to be a novel SMM marker representing whole-body SMM (18). Additionally, the T12 skeletal muscle index (T12 SMI) (i.e., T12 SMA/body height squared) and L3 SMI reportedly have a similar predictive value for 1-year mortality in older patients with trauma (19).

The present prospective cohort study aimed to answer the following three questions: (1) Is the T12 SMI a valid surrogate marker of whole-body SMM or trunk SMM in patients with advanced non-small cell lung cancer (NSCLC)? (2) Is chest CT-defined sarcopenia based on the T12 SMI associated with poor prognosis in this patient population? (3) Would the addition of handgrip strength and physical performance to chest CT-defined sarcopenia improve its predictive value for poor prognosis in this patient population?

## Materials and Methods

This study was conducted in accordance with the ethical principles outlined in the Declaration of Helsinki, and the study protocol was approved by the Biomedical Ethics Committee of West China Hospital, Sichuan University. All participants of this study provided signed informed consent.

### Patients

Adult patients diagnosed with advanced NSCLC at the Department of Oncology of Shangjin Nanfu Hospital, Sichuan University from August 2017 to May 2019 were prospectively and consecutively recruited. Adult patients who met the following criteria were included in the study: (1) pathologically confirmed stage IIIB or IV NSCLC based on the Union for International Cancer Control’s tumor–node–metastasis stage classification and (2) administration of first-line chemotherapy for the first time. However, patients (1) who received immune checkpoint inhibitor therapy, molecular targeted therapy, radiotherapy, or single-agent chemotherapy; (2) who had an implanted pacemaker; (3) who had a history of any other cancer type; (4) who had low-quality CT images or had any anatomical distortion (e.g. chest wall edema) or loss of any muscle mass area on CT images; and (5) who had visible edema were excluded from analysis.

### Clinical and Anthropometric Variables

The following clinical variables were recorded by trained nurses within 48 hours upon first admission to our department: age, sex, smoking status (never smoker or ever smoker), histologic type (adenocarcinoma, squamous cell carcinoma, or large cell carcinoma), cancer stage (stage IIIB or IV), Eastern Cooperative Oncology Group (ECOG) performance status (PS) score, number of chemotherapy courses, and chemotherapy regimens. Additionally, serum creatinine, serum albumin, and hemoglobin levels were measured within 24 hours after admission. The updated version of the Charlson comorbidity index (CCI) was used to evaluate the number and severity of important comorbidities, including chronic obstructive pulmonary disease, renal disease, any malignancy, and cerebrovascular disease (20). Data on comorbidities were directly collected from original medical records. The total CCI score is 24 points, whereas the score for ‘any malignancy’ is 2 points (20). Hence, a CCI score ≥3 indicated that a patient not only had NSCLC but also at least one other important comorbidity. Body height and weight were measured using standard methods. Body mass index (BMI) was calculated as body weight (kg) divided by body height squared (m^2^) and was categorized as follows: underweight, <20.0 kg/m^2^; normal, 20.0-24.9 kg/m^2^; and obese, ≥25 kg/m^2^ (4).

### CT Image Analysis

Chest CT scans were completed within 48 hours after admission for each participant using a 16-slice spiral CT scanner (Brilliance; Philips Healthcare, OH, USA) with a 5-mm slice thickness. Acquisition parameters were as follows: 100-140 kV, variable mA based on the patient’s body size, and detector collimation of 0.75-1.5 mm. Unenhanced cross-sectional CT images at the T12 level were analyzed using a dedicated segmentation software (Mimics version 21.0; Materialise, Leuven, Belgium) to evaluate the T12 SMA.

On a single CT image, all visualized skeletal muscles with a threshold of −29 to +150 HU, including the erector spinae, latissimus dorsi, rectus abdominis, obliquus externus, internus abdominis, and internal and external intercostal muscles, were segmented (12). The T12 SMI (cm^2^/m^2^) was calculated as the T12 SMA (cm^2^) divided by the body height squared (m^2^).

A trained observer (L.T.) who was blinded to patient outcomes during the analysis period segmented all CT images. To test the reliability of the T12 SMA determined by CT, a total of 30 participants were randomly selected from the cohort. To assess inter-observer reproducibility, another trained observer (S.H.) subsequently segmented the CT images again. Representative images are presented in **eFigure 1**.

### Bioelectrical Impedance Analysis (BIA) of Body Composition

On the day of the CT scan, trained nurses utilized a segmental multifrequency BIA device (InBody 770; Biospace, Korea) to measure each participant’s body composition, including the total lean body mass (LBM), trunk LBM, and appendicular LBM.

### Handgrip Strength Measurement

On the day of the CT scan, trained nurses also measured the handgrip strength to the nearest 0.1 kg using a digital grip dynamometer (EH101; Xiangshan Inc., Guangdong, China) in accordance with the recommendation of the Chinese National Physical Fitness Evaluation Standard (21). Three readings were obtained for each hand, and the highest value was recorded for analysis. Handgrip weakness was defined as handgrip strength <28 kg for men and <18 kg for women (11).

### Different Definitions of Sarcopenia

Sarcopenia was defined in the present study as CT-defined sarcopenia and Asian Working Group for Sarcopenia (AWGS)-defined sarcopenia.

(1) CT-defined sarcopenia (based on low SMM estimated by CT image analysis): As there currently exists no established cut-off value of the T12 SMI for determining low SMM, we set the optimal cut-off value for low SMM to predict overall survival (OS) as the diagnostic cut-off points for CT-defined sarcopenia using the maximally selected rank statistics method, as described by Lausen and Schumacher (22). This is a validated method for determining cut-off points that could optimally separate participants with respect to time to an event outcome (4, 23).

(2) AWGS-defined sarcopenia: According to the AWGS 2019 recommendation (11), patients with both low SMM and handgrip weakness (or low physical performance) are considered to have AWGS-defined sarcopenia, whereas patients with low SMM, handgrip weakness, and low physical performance are deemed to have AWGS-defined severe sarcopenia. The PS score is commonly used in routine clinical practice, with a PS score of 0-1 indicating good physical performance (24). In this study, low physical performance was defined as an ECOG PS score ≥2 points.

### Measurement of OS

OS was defined as the number of months elapsed from the date of initial recruitment to the date of death or last follow-up for each patient. Patients were followed up until their death or up to the last week of August 2020, at which time they were confirmed to be alive *via* telephone review and were subsequently censored.

### Statistical Analysis

Data analysis was conducted between October 3, 2020, and October 20, 2020. Histograms and the Shapiro–Wilk test were used to assess the distributions of continuous variables. All continuous variables were of normal distribution. Thus, continuous and categorical variables are expressed as mean (standard deviation) and number (percentage), respectively. Inter-observer validation of T12 SMI measurements was performed using interclass correlation coefficient analysis. Pearson’s correlation coefficient (r) and scatterplots with a linear regression model were employed to assess the association of the T12 SMI with total LBM, trunk LBM, appendicular LBM, BMI, handgrip strength, and the T12 SMA. Hazard ratios (HRs) of the T12 SMI and handgrip strength for predicting OS were evaluated using a Cox proportional hazards regression model with a restricted cubic spline function with three knots for men and women. Subsequently, we determined the cut-off values of the T12 SMI using the maximally selected rank statistics method (22). Using these cut-off values and the diagnostic criteria mentioned above, we defined patients with or without CT-defined sarcopenia and patients with or without AWGS-defined sarcopenia. Group differences were analyzed using one-way analysis of variance and chi-square test (or Fisher’s exact test), as appropriate.

OS curves for different groups were constructed using the Kaplan–Meier method and compared using the log-rank test. Additionally, univariate and multivariable analyses of OS were performed using Cox proportional hazards models, with the results presented as HRs with 95% confidence intervals (CI). Multivariable Cox Models 1, 2, and 3 evaluated the predictive value of CT-defined sarcopenia, AWGS-defined sarcopenia (as well as AWGS-defined severe sarcopenia), and physical performance, respectively, for predicting OS. In line with previous scientific literature (23, 25), the models were adjusted for age at baseline, sex, smoking status, histologic type, cancer stage, CCI, BMI groups, chemotherapy regimens, completion of at least four chemotherapy courses, and serum creatinine, serum albumin, and hemoglobin levels. Moreover, C-indexes were used to assess the discrimination performance of these models to predict OS (25), with c statistics of 0.5 indicating chance; 0.5-0.7, poor discrimination; 0.7-0.8, acceptable discrimination; 0.8-0.9, excellent discrimination; 0.9-0.99, outstanding discrimination; and 1.0, perfect prediction (23).

To assess the robustness of the results, a sensitivity analysis was conducted using the lowest quartile of the sex-specified T12 SMI to define low SMM, which was another widely employed method in previous studies (4). Subsequently, we accordingly redetermined CT-defined sarcopenia, AWGS-defined sarcopenia, and AWGS-defined severe sarcopenia, reperformed the univariate and multivariable analyses with the Cox proportional hazards models, and redrew the Kaplan–Meier curves.

Additionally, *a priori* subgroup analyses of multivariable Cox Models 1 and 2 were conducted according to age groups (<60 or ≥60 years), sex, cancer stage, histologic type, physical performance (0-1 points or 2 points), and number of chemotherapy courses (1-3 courses or ≥4 courses).

Statistical analyses were performed using R software version 3.5.3 (R Foundation for Statistical Computing, Vienna, Austria) and SPSS software version 26.0 (IBM Corp., Armonk, NY, USA). *P* values <.05 were considered to indicate statistical significance.

## Results

### Patient Characteristics

A total of 787 consecutive patients with advanced NSCLC were admitted to our department from August 2017 to May 2019. Of these patients, 82 refused to participate in this study. Furthermore, 15 patients with chest wall edema, 5 patients with low-quality CT images, 16 patients with missing data on handgrip strength, and 30 patients who received molecular targeted therapy were excluded. Accordingly, we included a total of 639 patients (410 men and 229 women) in the study. The median follow-up was 25 months (range: 15-36 months).

The mean age of the participants was 58.6 ± 8.9 years, with men being significantly older than women (mean age: 59.1 *vs*. 57.7 years, *P*=.049). The baseline characteristics of participants according to sex are summarized in **Table 1**. Overall, 294 (46%) and 345 (54.0%) patients had stage IIIB NSCLC and stage IV NSCLC, respectively. Because the histologic type, chemotherapy regimens, handgrip strength, and body composition variables were significantly different between men and women, we either adjusted for sex or split the data based on sex in the following analyses.

**Table 1:** Baseline characteristics of the study population according to sex.

Characteristics	Total (*n*=639)	Men (*n*=410)	Women (*n*=229)	*P* value^a^
Age, years, mean (SD)	58.6 (8.9)	59.1 (8.4)	57.7 (9.8)	.049
Age ≥60 years, *n* (%)	196 (30.7)	119 (29.0)	77 (33.6)	.227
Smoking status, *n* (%)				
Never smoker	311 (48.7)	97 (23.7)	214 (93.4)	<.001
Ever smoker	328 (51.3)	313 (76.3)	15 (6.6)	
Histologic type, *n* (%)				
Adenocarcinoma	394 (61.7)	213 (52.0)	181 (79.0)	<.001
Squamous cell carcinoma	201 (31.5)	173 (42.2)	28 (12.2)	
Large cell carcinoma	44 (6.9)	23 (5.9)	20 (8.7)	
Cancer stage, *n* (%)				
Stage IIIB	294 (46.0)	194 (47.3)	100 (43.7)	.375
Stage IV	345 (54.0)	216 (52.7)	129 (56.3)	
ECOG PS, *n* (%)				
0	385 (60.3)	249 (60.7)	126 (59.4)	.664
1	118 (18.5)	78 (19.0)	40 (17.5)	
≥ 2	136 (21.3)	93 (20.2)	53 (23.1)	
Body height, cm, mean (SD)	162.3 (7.7)	165.9 (6.0)	156.0 (6.3)	<.001
Body weight, kg, mean (SD)	60.9 (9.5)	63.4 (9.5)	56.6 (7.9)	<.001
BMI, kg/m^2^, mean (SD)	23.1 (3.1)	23.0 (3.0)	23.3 (3.2)	.264
BMI groups, *n* (%)				
Underweight (BMI <20)	383 (59.9)	66 (16.1)	29 (12.7)	.460
Normal weight (BMI 20-24.9)	95 (14.9)	143 (62.4)	143 (62.4)	
Obese (BMI ≥25)	161 (25.2)	104 (25.4)	57 (24.9)	
Charlson comorbidity index ≥3, *n* (%)	196 (30.7)	124 (30.2)	72 (31.4)	.753
Chemotherapy regimen, *n* (%)				
Pemetrexed + carboplatin/cisplatin	239 (37.4)	138 (33.7)	101 (44.1)	<.001
Docetaxel + carboplatin/cisplatin	234 (36.6)	144 (35.1)	90 (39.3)	
Gemcitabine + carboplatin/cisplatin	30 (4.7)	21 (5.1)	9 (3.9)	
Paclitaxel + carboplatin/cisplatin	136 (21.3)	107 (26.1)	29 (12.7)	
Patients who completed at least four chemotherapy courses, *n* (%)	498 (77.9)	325 (79.3)	173 (75.5)	.277
Serum creatinine, μmol/L, mean (SD)	72.2 (16.4)	77.5 (16.0)	62.6 (12.6)	<.001
Serum albumin, g/L, mean (SD)	41.9 (2.5)	42.3 (2.5)	41.3 (2.5)	<.001
Hemoglobin, g/L, mean (SD)	125.4 (23.1)	127.9 (23.4)	120.8 (21.8)	<.001
Body composition variables, mean (SD)				
T12 SMA, cm^2^	86.4 (18.2)	95.9 (14.4)	69.2 (9.8)	<.001
T12 SMI, cm^2^/m^2^	32.6 (5.9)	34.9 (5.4)	28.5 (4.4)	<.001
Total LBM, kg	26.5 (5.2)	28.5 (4.6)	23.0 (4.1)	<.001
Trunk LBM, kg	7.4 (1.9)	8.1 (2.0)	6.1 (0.3)	<.001
Appendicular LBM, kg	19.1 (4.2)	20.4 (3.8)	16.9 (3.9)	<.001
Handgrip strength, kg, mean (SD)	26.3 (6.9)	29.1 (5.8)	21.4 (5.9)	<.001
Handgrip weakness, *n* (%)	269 (42.1)	196 (47.8)	73 (31.9)	<.001
CT-defined sarcopenia, *n* (%)	160 (25.0)	102 (23.9)	58 (25.3)	.900
AWGS-defined sarcopenia, *n* (%)	141 (22.1)	97 (23.7)	44 (19.2)	.194
AWGS-defined severe sarcopenia, *n* (%)	42 (6.6)	30 (7.3)	12 (5.2)	.310

AWGS, Asian Working Group for Sarcopenia; BMI, body mass index; CT, computed tomography; ECOG PS, Eastern Cooperative Oncology Group performance status; LBM, lean body mass; SD, standard deviation; SMA, skeletal muscle cross-sectional area; SMI, skeletal muscle index (skeletal muscle area/height^2^); T12, 12^th^ thoracic vertebra. ^a^ Group differences were analyzed using one-way analysis of variance and chi-square test (or Fisher’s exact test), as appropriate.

### Association Between the T12 SMI, LBM, Handgrip Strength, and Physical Performance

T12 SMI measurements were highly reproducible between observers (**eFigure 2** in the **Supplement**). Considering that the T12 SMI is not a classical surrogate of muscle mass, we analyzed the association of the T12 SMI with BIA-derived LBM, handgrip strength, and physical performance. As shown in **eFigure 3** in the **Supplement**, the T12 SMI was highly correlated with trunk LBM and handgrip strength (*r*=0.78, *P*<.001 and *r*=0.70, *P*<.001, respectively) and was moderately correlated with total LBM (*r*=0.57, *P*<.001). Scatterplots with linear regression for these variables are presented in **eFigures 4A–E** in the **Supplement**. Moreover, both male and female patients with low physical performance (PS score ≥2) had a significantly lower T12 SMI (**eFigure 4F** in the **Supplement**).

### Impact of the T12 SMI and Handgrip Strength on OS

**Figure 1:**
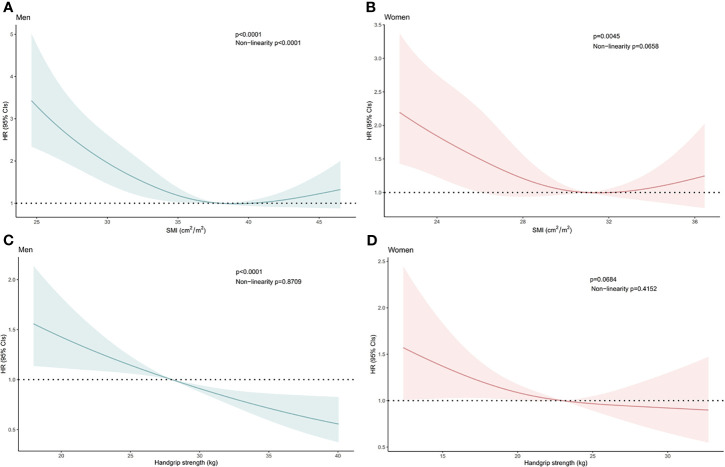
Hazard Ratios of Overall Survival Related to **(A)** the Skeletal Mass Index at the 12th Thoracic Vertebra Level (T12 SMI) in Men, **(B)** the T12 SMI in Women, **(C)** Handgrip Strength in Men, and **(D)** Handgrip Strength in Women. Hazard ratios of the T12 SMI and handgrip strength were estimated as continuous data using a restricted cubic spline function with three knots. Bold lines represent the curves for the estimated hazard ratios related to the T12 SMI or handgrip strength in men and women. Shadowed areas indicate the corresponding 95% confidence intervals. CI, confidence interval; HR, hazard ratio.

The T12 SMI was a significant factor for OS in men (*P*<.001, **Figure 1A**) and women (*P*=.005, **Figure 1B**). Handgrip strength was also a significant factor for OS in men (*P*<.001, **Figure 1C**). Furthermore, lower handgrip strength exhibited a tendency toward poor prognosis in women (*P*=.068, **Figure 1D**).

### Prevalence of Sarcopenia in Participants

**Figure 2:**
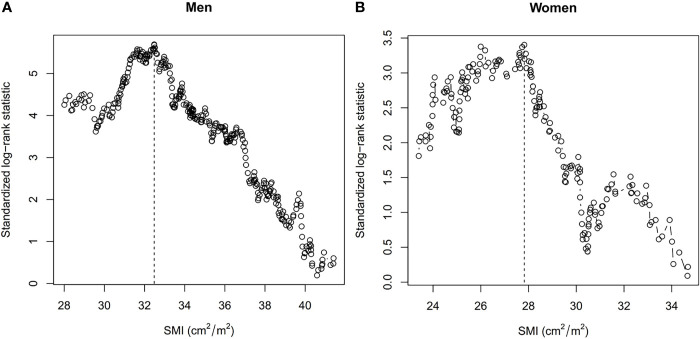
Cut-off Values of the Skeletal Mass Index at the 12^th^ Thoracic Vertebra Level (T12 SMI) in **(A)** Men and **(B)** Women Using Maximally Selected Rank Statistics. Dashed lines show the cut-off values. SMI, skeletal mass index.

Based on maximally selected rank statistics calculation, we set the cut-off values of the T12 SMI as 32.48 cm^2^/m^2^ for men and 27.82 cm^2^/m^2^ for women (**Figure 2**). At baseline, 160 (25.0%), 141 (22.1%), and 42 (6.6%) patients had CT-defined sarcopenia, AWGS-defined sarcopenia, and AWGS-defined severe sarcopenia, respectively. The prevalence of CT-defined sarcopenia, AWGS-defined sarcopenia, and AWGS-defined severe sarcopenia were not significantly different between men and women (**Table 1**). The clinicopathological characteristics of participants according to CT-defined sarcopenia or AWGS-defined sarcopenia are summarized in **eTable 1** in the **Supplement**.

### Impact of CT-Defined Sarcopenia, Handgrip Weakness, Poor Physical Performance, and AWGS-Defined Sarcopenia on OS

**Figure 3:**
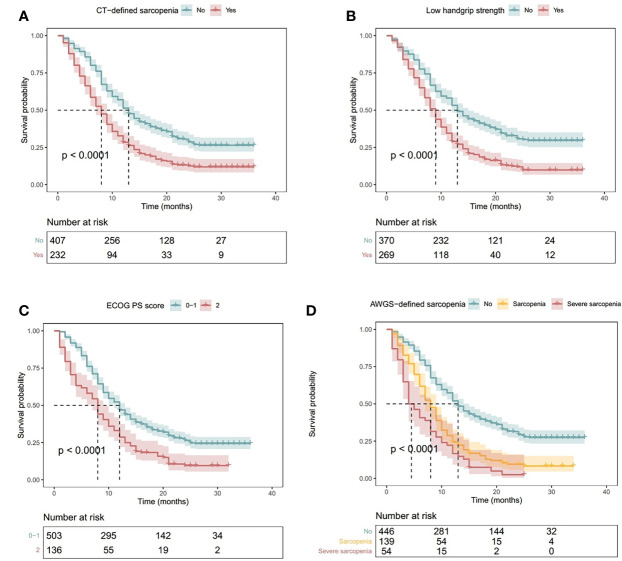
Kaplan–Meier Curves Illustrating Overall Survival in Patients with **(A)** CT-Defined Sarcopenia, **(B)** Handgrip Weakness, **(C)** Low Physical Performance (ECOG PS Score ≥2), and **(D)** AWGS-Defined Sarcopenia or Severe Sarcopenia. *P* values indicate the results of the log-rank test. Shadowed areas indicate 95% confidence intervals. AWGS, Asian Working Group for Sarcopenia; CT, computed tomography; ECOG PS: Eastern Cooperative Oncology Group performance status.

A total of 488 (76.4%) patients died during the study period. Patients with CT-defined sarcopenia had a shorter OS than patients without CT-defined sarcopenia (**Figure 3A**, log-rank *P*<.001). Similarly, patients exhibiting handgrip weakness had a shorter OS than those with normal handgrip strength (**Figure 3B**, log-rank *P*<.001). Patients with low physical performance had a shorter OS than those with normal physical performance (**Figure 3C**, log-rank *P*<.001). Moreover, patients with AWGS-defined sarcopenia had a shorter OS than those without AWGS-defined sarcopenia, whereas patients with AWGS-defined severe sarcopenia had the worst prognosis (**Figure 3D**, log-rank *P*<.001).

Univariate and multivariable analyses with Cox proportional hazards models were performed to identify independent factors associated with OS (**Table 2**). After adjustment for the same confounders, CT-defined sarcopenia (Model 1: HR, 2.00; 95% CI, 1.65-2.43) and AWGS-defined sarcopenia (Model 2: HR, 2.00; 95% CI, 1.59-2.49) were associated with poor prognosis. AWGS-defined severe sarcopenia indicated a higher risk of poor prognosis (Model 2: HR, 3.01; 95% CI, 2.21-4.09). All these indicators were more strongly associated with poor prognosis than low physical performance (PS score ≥2) (Model 3: HR, 1.37; 95% CI, 1.10-1.73).

**Table 2:** Median survival and univariate and multivariable analyses for predictors of overall survival.

Variables	No. of Patients	No. of Deaths	Survival (Months)		Univariate Analysis			Multivariable Analysis			Multivariable Analysis			Multivariable Analysis		
								Model 1			Model 2			Model 3		
			Median	95% CI	HR	95% CI	*P* Value	HR	95% CI	*P* Value	HR	95% CI	*P* Value	HR	95% CI	*P* Value
CT-defined sarcopenia																
No	407	287	13.0	11.8-14.2	1	Reference		1	Reference		–	–	–	–	–	–
Yes	232	201	8.0	6.9-9.1	1.80	1.50-2.15	<.001	2.00	1.65-2.43	<.001	–	–	–	–	–	–
AWGS-defined sarcopenia																
No sarcopenia	446	311	13.0	11.7-14.3	1	Reference		–	–	–	1	Reference		–	–	–
Sarcopenia	139	125	8.0	6.9-9.1	2.00	1.62-2.46	<.001	–	–	–	2.00	1.59-2.49	<.001	–	–	–
Severe sarcopenia	54	52	4.0	2.3-5.7	2.99	2.22-4.03	<.001	–	–	–	3.01	2.21-4.09	<.001	–	–	–
ECOG PS																
0-1	503	367	12.0	10.8-13.2	1	Reference		–	–	–	–	–	–	1	Reference	
≥ 2	136	121	8.0	6.8-9.2	1.78	1.44-2.18	<.001	–	–	–	–	–	–	1.37	1.10-1.71	.006
Age per year	–	–	–	–	1.08	1.07-1.09	<.001	1.09	1.08-1.11	<.001	1.09	1.08-1.11	<.001	1.08	1.07-1.10	<.001
Sex																
Male	410	323	10.0	9.0-11.0	1	Reference		1	Reference		1	Reference		1	Reference	
Female	229	165	11.0	9.0-13.0	0.89	0.74-1.07	.208	0.77	0.59-1.02	.063	0.83	0.63-1.09	.184	0.80	0.61-1.05	.110
Smoking status																
Never smoker	311	238	11.0	9.7-12.3	1	Reference		1	Reference		1	Reference		1	Reference	
Ever smoker	328	250	11.0	9.6-12.4	1.05	0.88-1.26	.593	1.17	0.92-1.49	.226	1.22	0.96-1.55	.117	1.13	0.89-1.44	.306
Histologic type																
Adenocarcinoma	394	303	10.0	8.9-11.1	1	Reference		1	Reference		1	Reference		1	Reference	
Squamous cell carcinoma	201	146	13.0	11.5-14.5	0.87	0.71-1.07	.181	0.91	0.73-1.15	.494	0.92	.073-1.16	.485	0.89	0.70-1.12	.303
Large cell carcinoma	44	39	5.0	1.8-8.3	1.95	1.39-2.72	<.001	1.55	1.09-2.20	.023	1.42	1.01-2.02	.049	1.58	1.12-2.24	.010
Cancer stage																
Stage IIIB	294	203	17.0	14.5-19.5	1	Reference		1	Reference		1	Reference		1	Reference	
Stage IV	345	285	8.0	7.3-8.7	1.92	1.60-2.30	<.001	2.81	2.30-3.42	<.001	2.86	2.34-3.49	<.001	2.82	2.31-3.44	<.001
Charlson comorbidity index																
0	443	325	11.0	9.8-12.2	1	Reference		1	Reference		1	Reference		1	Reference	
≥1	196	163	10.0	8.5-11.5	1.23	1.02-1.48	.034	1.17	0.96-1.43	.187	1.18	0.97-1.44	.099	1.16	0.95-1.42	.144
BMI group																
Underweight	95	75	10.0	8.3-11.8	1.14	0.89-1.47	.303	0.92	0.70-1.21	.581	0.90	0.69-1.19	.472	1.05	0.80-1.37	.746
Normal	383	297	10.0	8.8-11.2	1	Reference		1	Reference		1	Reference		1	Reference	
Obese	161	116	12.0	10.6-13.4	0.80	0.65-0.99	.045	0.91	0.72-1.14	.444	0.84	0.67-1.05	.128	0.83	0.67-1.04	.098
Chemotherapy regimen																
Pemetrexed + carboplatin/cisplatin	239	184	10.0	8.8-11.2	1	Reference		1	Reference		1	Reference		1	Reference	
Docetaxel + carboplatin/cisplatin	234	179	12.0	10.2-13.8	0.93	0.76-1.14	.476	1.05	0.84-1.32	.669	1.05	0.84-1.32	.656	1.07	0.86-1.34	.551
Gemcitabine + carboplatin/cisplatin	30	26	8.0	5.7-10.3	1.36	0.90-2.05	.142	0.97	0.64-1.48	.896	0.99	0.65-1.50	.949	1.12	0.74-1.71	.587
Paclitaxel + carboplatin/cisplatin	136	99	11.0	8.9-13.1	0.91	0.71-1.16	.451	1.02	0.78-1.31	.912	1.01	0.78-1.31	.945	1.00	0.78-1.30	.967
Patients who completed at least four chemotherapy courses																
No	141	120	9.0	8.1-9.9	1	Reference		1	Reference		1	Reference		1	Reference	
Yes	498	368	12.0	10.9-13.1	0.72	0.59-0.89	.002	0.78	0.59-0.97	.028	0.75	0.58-0.96	.021	0.82	0.64-1.05	.111
Serum creatinine per SD	–	–	–	–	1.09	1.00-1.18	.051	0.97	0.87-1.08	.569	0.98	0.88-1.09	.740	0.97	0.87-1.08	.606
Serum albumin per SD	–	–	–	–	1.06	0.97-1.16	.217	1.07	0.96-1.18	.305	1.07	0.96-1.18	.227	1.09	0.98-1.21	.119
Hemoglobin per SD	–	–	–	–	1.03	0.95-1.12	.501	1.03	0.93-1.13	.548	1.01	0.92-1.12	.797	1.00	0.91-1.10	.961
C statistic^a^								0.72	0.69-0.74		0.76	0.75-0.78		0.69	0.67-0.72	

AWGS, Asian Working Group for Sarcopenia; BMI, body mass index; CT, computed tomography; ECOG PS, Eastern Cooperative Oncology Group performance status; LBM, lean body mass; SD, standard deviation.

a A c statistic of 0.5 indicates chance; 0.5-0.7, poor discrimination; 0.7-0.8, acceptable discrimination; 0.8-0.9, excellent discrimination; 0.9-0.99, outstanding discrimination; and 1.0, perfect prediction.

The c statistics of the multivariable Cox Models were compared (**Table 2**). The c statistics were 0.72 (95% CI, 0.69-0.74) for Model 1 and 0.76 (95% CI, 0.75-0.78) for Model 2, indicating moderate discrimination for OS. Model 2 was better than Model 1 in discriminating OS. The c statistic for Model 3 was 0.69 (95% CI, 0.67-0.72), indicating poor discrimination for OS.

### Sensitivity Analysis

To examine the robustness of our results, we performed an *a priori* sensitivity analysis by defining low SMM as the lowest quartile of the sex-specified study population and subsequently redetermined the proportions of CT-defined sarcopenia, AWGS-defined sarcopenia, and AWGS-defined severe sarcopenia accordingly. Afterwards, we reperformed univariate and multivariable analyses with Cox proportional hazards models (**eTable 2** in the **Supplement**) and redrew the Kaplan–Meier curves (**eFigure 5** in the **Supplement**). The results remained almost identical.

### Subgroup Analyses

**Figure 4:**
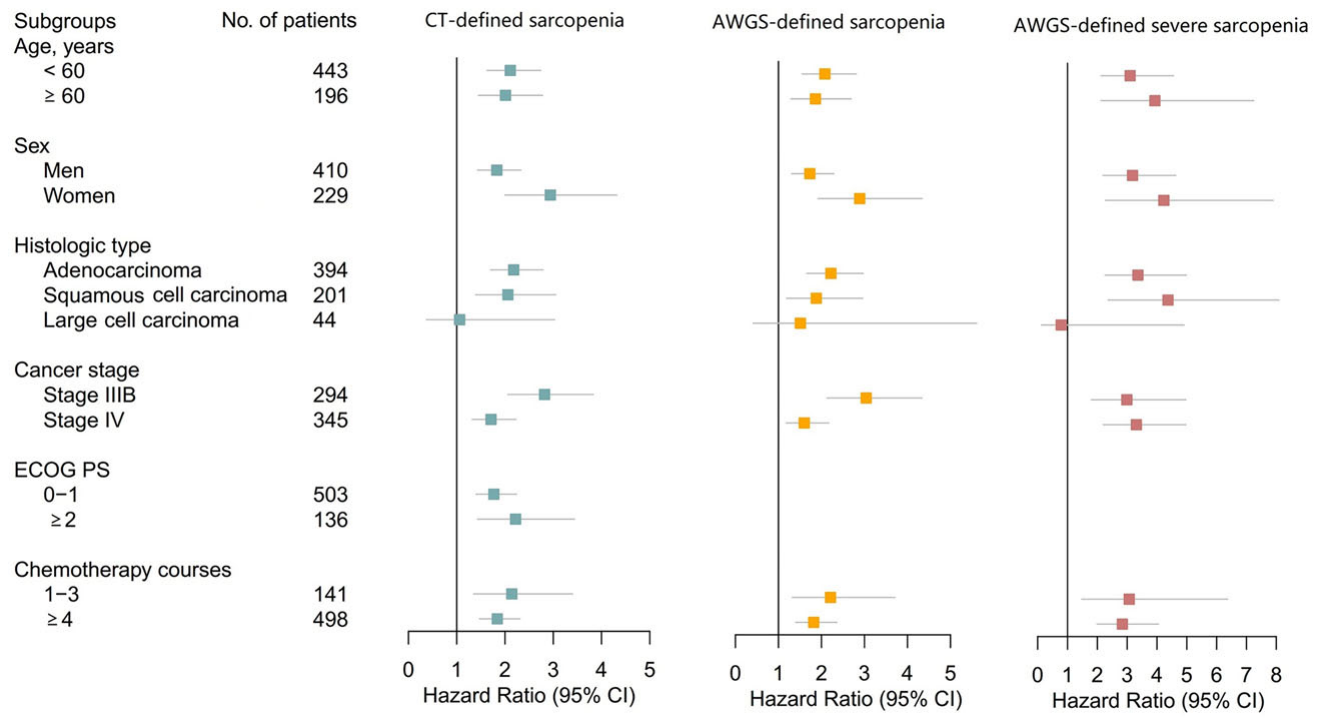
Forest Plots Illustrating Subgroup Analyses According to Age, Sex, Histologic Type, Cancer Stage, ECOG PS, and Chemotherapy Courses. Subgroup analysis according to ECOG PS groups was not performed for AWGS-defined sarcopenia and severe sarcopenia because ECOG PS scores are a component of AWGS-defined sarcopenia and severe sarcopenia. AWGS, Asian Working Group for Sarcopenia; CI, confidence interval; CT, computed tomography; ECOG PS, Eastern Cooperative Oncology Group performance status.

Lastly, we performed *a priori* subgroup analyses of multivariable Cox Models 1 and 2. Most of these analyses revealed that CT-defined sarcopenia, AWGS-defined sarcopenia, and AWGS-defined severe sarcopenia were all significantly associated with poor prognosis (**Figure 4**). However, none of these conditions were significantly associated with poor prognosis in patients with large cell lung cancer.

## Discussion

The present study attempted to fill the knowledge gap between the fields of oncology and surgery and the fields of geriatric and internal medicine with respect to the definition of sarcopenia. To our knowledge, this is the first prospective cohort study that directly compared the prognostic values of CT-defined sarcopenia (i.e., low SMM) and AWGS-defined sarcopenia and severe sarcopenia (i.e., combination of low SMM, handgrip weakness, and/or low physical performance) in patients with lung cancer. We determined that CT-defined sarcopenia based on the T12 SMI derived from a single-slice chest CT image was a better prognostic factor for OS than the conventional PS score. Furthermore, the addition of handgrip strength and the PS score to CT-defined sarcopenia could further improve OS discrimination in our study population.

In the present study, CT-defined sarcopenia based on the T12 SMI was associated with poor prognosis in patients with advanced NSCLC, even in those with normal PS scores. This finding highlights the crucial role of chest CT-defined sarcopenia in NSCLC. Numerous studies have proven that sarcopenia is a useful prognostic factor for predicting OS, disease-free survival, and adverse events of various treatments in patients with lung cancer; nevertheless, the majority of these studies had a retrospective design and were based on the L3 SMI or L3 psoas muscle mass derived from abdominal CT images (19, 26–28). We failed to retrieve any study addressing the T12 SMI and prognosis in lung cancer. However, we identified two small retrospective studies that examined the prognostic value of the change in the T12 SMA before and after surgery in patients with NSCLC (29, 30). Both studies were from the same research team, and their results confirmed that the postoperative decrease in the T12 SMA was associated with poor prognosis (29, 30). Similarly, another retrospective study showed that the T12 SMA automatically derived from CT images was associated with all-cause mortality in a multicenter cohort of older community-dwelling men (31). These findings altogether suggest that further validation of the T12 SMI as a promising surrogate marker of whole-body SMM and a good prognostic factor is warranted in oncological and geriatric research.

Our study revealed that the combination of CT-defined sarcopenia, handgrip weakness, and/or low physical performance (i.e. AWGS-defined sarcopenia or AWGS-defined severe sarcopenia) could further improve OS discrimination. Few studies have addressed a similar issue in the literature. Burtin et al. recently published a prospective study that evaluated handgrip strength and fat-free mass (a surrogate indicator of BIA-derived SMM) for prognostic prediction in patients with stage I-II NSCLC treated with curative-intent radiotherapy (23). They concluded that handgrip weakness and low fat-free mass were independent prognostic factors for OS and that patients with both conditions exhibited worse prognosis (23). While their findings were in line with ours, they did not consider low physical performance as a component of sarcopenia. Our study employed a PS score ≥2 to define low physical performance, which is not recommended by either AWGS 2019 (11) or EWGSOP2 (10). Both guidelines recommend the use of the Short Physical Performance Battery, usual gait speed, or 5-time chair stand test for physical performance assessment. However, these tests are not routinely performed in clinical practice and are not only time-consuming but also labor-intensive. Our study indicated that a PS score ≥2, a convenient indicator, could be used to define low physical performance in patients with lung cancer.

### Clinical Implications

Because chest CT scans are always available for patients with lung cancer, the clinical utility of chest CT scans, rather than abdominopelvic CT scans, is important in sarcopenia assessment. Furthermore, various organizations such as the US Preventive Services Task Force (32) and the Chinese Society of Clinical Oncology (33) have recommended low-dose chest CT scans for lung cancer screening in individuals with risk factors. The opportunistic utility of chest CT scans for screening lung cancer has been increasing to identify other diseases, such as chronic obstructive pulmonary disease and osteoporosis (34–36). Similarly, assessment of muscle health would be another opportunistic utility of these screening CT images.

The addition of handgrip strength and the PS score to chest CT-defined sarcopenia could further provide prognostic information on advanced NSCLC. Considering that the PS score is commonly obtained during routine oncological evaluation and that handgrip strength can be easily and repeatedly measured throughout cancer management without increasing any burden to patients, it is reasonable to use the combination of low SMM (derived from chest CT), handgrip weakness, and low physical performance (PS score ≥2) to define sarcopenia in the oncological research field.

### Limitations

Our study also has some limitations. First, our study was conducted at a single center; hence, the generalizability of our results seems to be limited. Second, in general, our study population might not be representative of patients with advanced NSCLC owing to potential referral bias. Third, we used a segmental multifrequency BIA device instead of dual-energy X-ray absorptiometry to estimate LBM. Nevertheless, according to a recent study, LBM measured by segmental multifrequency BIA has good agreement with dual-energy X-ray absorptiometry in ambulatory individuals (35). Fourth, we did not evaluate some important outcomes, including disease-specific mortality, quality of life, functional decline, and the incidence of adverse events related to chemotherapy.

## Conclusions

The SMI derived from a single-slice chest CT image at the T12 level was a valid surrogate marker of whole-body muscle mass. CT-defined sarcopenia based on the T12 SMI and a PS score ≥2 were independent prognostic factors for OS in patients with advanced NSCLC who received first-line chemotherapy. CT-defined sarcopenia was a better prognostic factor for OS than the conventional prognostic factor (PS score ≥2) in this patient population. The addition of handgrip strength and the PS score to chest CT-defined sarcopenia could further provide prognostic information on advanced NSCLC. Sarcopenia research in oncology should focus not only on muscle mass but also on muscle strength and function.

## Data Availability Statement

The raw data supporting the conclusions of this article will be made available by the authors, without undue reservation.

## Ethics Statement

The studies involving human participants were reviewed and approved by the Biomedical Ethics Committee of West China Hospital, Sichuan University. The patients/participants provided their written informed consent to participate in this study.

## Author Contributions

MY had full access to all data and takes responsibility for the integrity of the data and the accuracy of the data analysis. Concept and design:MY and WL. Acquisition and analysis of CT images: SH and LT. Acquisition of clinical data: LX and JW. Data analysis and interpretation: MY, LT, and DL. Drafting of the manuscript: MY and LT. Critical revision of the manuscript for important intellectual content: All authors. Acquisition of funding: MY and WL. Administrative, technical, or material support: MY and WL. Supervision: MY and LX. All authors contributed to the article and approved the submitted version.

## Funding

This research was supported by the Post-Doctor Research Project, West China Hospital, Sichuan University (2018HXBH071) and the K&D Program of Sichuan Science and Technology Department (2020YFS0573).

## Conflict of Interest

The authors declare that the research was conducted in the absence of any commercial or financial relationships that could be construed as a potential conflict of interest.

## Publisher’s Note

All claims expressed in this article are solely those of the authors and do not necessarily represent those of their affiliated organizations, or those of the publisher, the editors and the reviewers. Any product that may be evaluated in this article, or claim that may be made by its manufacturer, is not guaranteed or endorsed by the publisher.
