# Supplementary material for: Factors That Improve Chest Computed Tomography-Defined Sarcopenia Prognosis in Advanced Non-Small Cell Lung Cancer
Source: Front Oncol. 2021 Oct 1;11:754975. doi: 10.3389/fonc.2021.754975 (PMC8517486; doi:10.3389/fonc.2021.754975)
Supplement: Supplementary file 1 [file DataSheet_1.pdf]

Online-Only Supplements

eTable 1. Clinicopathological Characteristics of Patients According to CT-Defined Sarcopenia or AWGS-Defined Sarcopenia and Severe Sarcopenia

eTable 2. Median Survival and Univariate and Multivariate Analyses for Predictors of Overall Survival (Sensitivity Analysis)

eFigure 1. Cross-Sectional Computed Tomography (CT) Images at the 12th Thoracic Vertebra Level Used for the Quantification of the Skeletal Muscle Area (T12 SMA)

eFigure 2. Inter-observer Validation Using Interclass Correlation Coefficient Analysis

eFigure 3. Correlation Matrix of Total Lean Body Mass (LBM), Trunk LBM, Handgrip Strength (HGS), Skeletal Mass Index at the 12th Thoracic Vertebra Level (T12 SMI), Body Mass Index (BMI), and Skeletal Mass Area at the 12th Thoracic Vertebra Level (T12 SMA)

eFigure 4. Correlations of T12 SMI with (A) Total Lean Body Mass (LBM), (B) Trunk LBM, (B) Appendicular LBM, (D) BMI, and (E) Handgrip Strength

eFigure 5. Sensitivity Analysis of Kaplan–Meier Curves Showing Overall Survival in Patients with (A) CT-Defined Sarcopenia and (B) AWGS-Defined Sarcopenia or Severe Sarcopenia

eTable 1. Clinicopathological Characteristics of Patients According to CT-Defined Sarcopenia or AWGS-Defined Sarcopenia and Severe Sarcopenia

	CT-Defined Sarcopenia			AWGS-Defined Sarcopenia			
**Characteristics**	**No (*n*=407)**	**Yes (*n*=232)**	***P* Value [a]**	**No (*n*=446)**	**Sarcopenia (*n*=139)**	**Severe Sarcopenia (*n*=54)**	***P* Value [a]**
Age, years, mean (SD)	58.6 (9.0)	58.6 (8.9)	.992	58.2 (9.0)	59.1 (8.6)	60.6 (8.9)	.128
Age ≥60 years, *n* (%)	125 (30.7)	71 (30.6)	.977	131 (29.4)	48 (34.5)	17 (31.5)	.510
Women, *n* (%)	130 (31.9)	99 (42.7)	.007	160 (35.9)	53 (38.1)	16 (29.6)	.543
Ever smoker, *n* (%)	219 (53.8)	109 (47.0)	.097	227 (50.9)	71 (51.1)	30 (55.6)	.809
Histologic type, *n* (%)							
Adenocarcinoma	248 (60.9)	146 (62.9)	.139	277 (62.1)	84 (60.4)	33 (61.1)	.237
Squamous cell carcinoma	136 (33.4)	65 (28.0)		145 (32.5)	41 (29.5)	15 (27.8)	
Large cell carcinoma	23 (5.7)	21 (9.1)		24 (5.4)	14 (10.1)	6 (11.1)	
Cancer stage, *n* (%)							
Stage IIIB	190 (46.7)	104 (44.8)	.651	209 (46.9)	62 (44.6)	23 (42.6)	.781
Stage IV	217 (53.3)	128 (55.2)		237 (53.1)	77 (55.4)	31 (57.4)	
ECOG PS, *n* (%)							
0	271 (66.6)	114 (49.1)	<.001	306 (68.6)	79 (56.8)	0 (0)	<.001
1	75 (18.4)	43 (18.5)		79 (17.7)	39 (28.1)	0 (0)	
≥2	61 (15.0)	75 (32.3)		61 (13.7)	21 (15.1)	54 (100)	
BMI groups, *n* (%)							
Underweight (BMI <20)	39 (9.6)	56 (24.1)	<.001	43 (9.6)	37 (26.6)	15 (27.8)	<.001
Normal weight (BMI of 20-24.9)	236 (58.0)	147 (63.4)	<.001	268 (60.1)	85 (61.2)	30 (55.6)	
Obesity (BMI ≥25)	132 (32.4)	29 (12.5)		135 (30.3)	17 (12.2)	9 (16.7)	
Charlson comorbidity index ≥3, *n* (%)	121 (29.7)	75 (32.3)	.493	130 (29.1)	47 (33.8)	19 (35.2)	.438
Chemotherapy regimens, *n* (%)							
Pemetrexed + carboplatin/cisplatin	151 (37.1)	88 (37.9)	.408	167 (37.4)	52 (37.4)	20 (37.0)	.387
Docetaxel + carboplatin/cisplatin	151 (37.1)	83 (35.8)		169 (37.9)	45 (32.4)	20 (37.0)	
Gemcitabine + carboplatin/cisplatin	15 (3.7)	15 (6.5)		16 (3.6)	9 (6.5)	5 (9.3)	
Paclitaxel + carboplatin/cisplatin	90 (22.1)	46 (19.8)		94 (21.1`)	33 (23.7)	9 (16.7)	
Patients who completed at least four chemotherapy	318 (78.1)	180 (77.6)	.873	346 (77.6)	109 (78.4)	43 (79.6)	.931

courses, *n* (%)							
BMI in men, kg/m^2, mean (SD)	23.6 (2.9)	21.8 (3.0)	<.001	23.6 (2.9)	21.6 (2.9)	21.9 (3.2)	<.001
BMI in women, kg/m^2, mean (SD)	24.2 (3.1)	22.1 (2.9)	<.001	23.7 (3.1)	22.4 (3.4)	22.4 (2.4)	.018
Serum albumin in men, g/L, mean (SD)	42.3 (2.4)	42.3 (2.6)	.887	42.3 (2.4)	42.1 (2.7)	42.6 (2.4)	.601
Serum albumin in women, g/L, mean (SD)	41.1 (2.4)	41.5 (2.6)	.228	41.2 (2.5)	41.6 (2.7)	41.9 (2.2)	.464
Hemoglobin in men, g/L, mean (SD)	128.4 (22.0)	125.0 (25.9)	.073	129.1 (22.4)	122.6 (26.3)	131.3 (22.9)	.053
Hemoglobin in women, g/L, mean (SD)	123.5 (18.9)	117.2 (24.7)	.031	121.6 (20.4)	118.1 (27.4)	121.5 (11.4)	.595
Body composition variables							
T12 SMA in men, cm^2, mean (SD)	102.7 (11.6)	81.9 (8.1)	<.001	102.2 (11.7)	81.6 (8.3)	81.2 (8.2)	<.001
T12 SMA in women, cm^2, mean (SD)	75.2 (7.5)	61.4 (6.5)	<.001	73.2 (8.3)	60.0 (6.5)	60.3 (6.2)	<.001
T12 SMI in men, cm^2/m^2, mean (SD)	37.7 (3.9)	29.2 (2.7)	<.001	37.5 (4.1)	29.1 (2.7)	29.0 (2.9)	<.001
T12 SMI in women, cm^2/m^2, mean (SD)	31.5 (3.0)	24.6 (2.1)	<.001	30.4 (3.7)	24.2 (2.4)	24.4 (1.6)	<.001
Total LBM in men, kg, mean (SD)	29.5 (4.6)	26.4 (3.8)	<.001	29.5 (4.6)	26.1 (3.8)	26.4 (3.7)	<.001
Total LBM in women, kg, mean (SD)	23.9 (4.0)	21.8 (4.0)	<.001	23.5 (4.1)	21.5 (2.7)	22.9 (5.1)	.010
Trunk LBM in men, kg, mean (SD)	8.9 (1.9)	6.5 (1.1)	<.001	8.8 (1.9)	6.5 (1.0)	6.5 (1.3)	<.001
Trunk LBM in women, kg, mean (SD)	6.5 (1.0)	5.5 (0.5)	<.001	6.3 (1.0)	5.6 (0.5)	5.5 (0.6)	<.001
Appendicular LBM in men, kg, mean (SD)	20.7 (4.0)	19.8 (3.4)	.039	20.7 (4.0)	19.7 (3.4)	19.8 (3.2)	.063
Appendicular LBM in women, kg, mean (SD)	17.4 (3.9)	16.3 (3.8)	.030	17.2 (3.9)	15.9 (3.5)	17.4 (4.7)	.127
Handgrip strength in men, kg, mean (SD)	31.0 (5.7)	25.1 (3.6)	<.001	30.9 (5.6)	25.0 (3.1)	23.9 (4.1)	<.001
Handgrip strength in women, kg, mean (SD)	23.8 (5.4)	18.3 (4.9)	<.001	23.6 (5.1)	16.9 (4.4)	14.6 (3.2)	<.001
Handgrip weakness, *n* (%)	97 (23.8)	172 (74.1)	<.001	97 (21.7)	118 (84.9)	54 (100)	<.001

Abbreviations: AWGS, Asian Working Group for Sarcopenia; BMI, body mass index; CT, computed tomography; ECOG PS, Eastern Cooperative Oncology Group performance status; LBM, lean body mass; SD, standard deviation; SMA, skeletal muscle cross-sectional area; SMI, skeletal muscle index (skeletal muscle cross-sectional area/height^2); T12, 12th thoracic vertebra.

[a]Group differences were analyzed using one-way analysis of variance and chi-square test (or Fisher's exact test), as appropriate.

eTable 2. Median Survival and Univariate and Multivariate Analyses for Predictors of Overall Survival (Sensitivity Analysis)[a]

			Survival (Months)		Univariate Analysis			Multivariate Analysis Model 1			Multivariate Analysis Model 2		
Variables	No. of Patients	No. of Deaths	Median	95% CI	HR	95% CI	*P* Value	HR	95% CI	*P* Value	HR	95% CI	*P* Value
CT-defined sarcopenia													
No	479	348	12.0	12.0-14.0	1	Reference		1	Reference		-	-	-
Yes	160	140	7.0	7.0-8.0	1.93	1.59-2.36	<.001	2.12	1.71-2.63	<.001	-	-	-
AWGS-defined sarcopenia													
Without sarcopenia	498	359	13.0	12.0-14.0	1	Reference		-	-	-	1	Reference	
Sarcopenia	99	89	8.0	7.0-9.0	1.98	1.56-2.50	<.001	-	-	-	1.85	1.43-2.38	<.001
Severe sarcopenia	42	40	4.0	3.0-6.0	3.76	2.69-5.24	<.001	-	-	-	4.19	2.95-5.93	<.001
Age per year	-	-	-	-	1.08	1.07-1.09	<.001	1.09	1.08-1.11	<.001	1.09	1.08-1.10	<.001
Sex													
Men	410	323	10.5	9.5-12.0	1	Reference		1	Reference		1	Reference	
Women	229	165	11.0	9.0-13.0	0.89	0.74-1.07	.208	0.83	0.63-1.47	.193	0.84	0.64-1.11	.226
Smoking status													
Never smoker	311	238	11.0	9.0-12.0	1	Reference		1	Reference		1	Reference	
Ever smoker	328	250	11.0	10.0-13.0	1.05	0.88-1.26	.593	1.15	0.91-1.47	.248	1.17	0.92-1.50	.206
Histologic type													
Adenocarcinoma	394	303	10.5	9.0-12.0	1	Reference		1	Reference		1	Reference	
Squamous cell carcinoma	201	146	13.0	10.0-14.0	0.87	0.71-1.07	.181	0.95	0.75-1.20	.945	0.94	0.74-1.18	.572
Large cell carcinoma	44	39	5.5	3.0-8.5	1.95	1.39-2.72	<.001	1.55	1.08-2.19	.015	1.52	1.07-2.16	.020
Cancer stage													
Stage IIIB	294	203	16.0	14.0-17.0	1	Reference		1	Reference		1	Reference	
Stage IV	345	285	9.0	7.0-11.0	1.92	1.60-2.30	<.001	2.75	2.25-3.36	<.001	2.73	2.23-3.33	<.001
ECOG PS													
0-1	503	367	12.0	11.0-13.0	1	Reference		1	Reference		-	-	-
≥2	136	121	8.0	6.0-9.0	1.78	1.44-2.18	<.001	1.34	1.08-1.68	.008	-	-	-
Charlson comorbidity index													
0	443	325	11.0	10.0-12.0	1	Reference		1	Reference		1	Reference	
≥3	196	163	10.0	9.0-12.0	1.23	1.02-1.48	.034	1.19	0.97-1.46	.088	1.23	1.01-1.50	.042
BMI groups													
Underweight	95	75	10.0	8.0-12.0	1.14	0.89-1.47	.303	0.93	0.70-1.22	.582	0.90	0.68-1.18	.439
Normal weight	383	86	10.0	9.0-11.5	1	Reference		1	Reference		1	Reference	

Overweight or obesity	161	116	12.0	12.0-15.0	0.80	0.65-0.99	.045	0.94	0.75-1.17	.560	0.90	0.72-1.12	.343
Chemotherapy regimens													
Pemetrexed + carboplatin/cisplatin	239	184	10.0	9.0-12.0	1	Reference		1	Reference		1	Reference	
Docetaxel + carboplatin/cisplatin	234	179	12.0	10.0-13.0	0.93	0.76-1.14	.476	0.97	0.78-1.20	.743	0.98	0.79-1.22	.860
Gemcitabine + carboplatin/cisplatin	30	26	8.5	5.0-11.5	1.36	0.90-2.05	.142	0.96	0.63-1.47	.851	1.02	0.67-1.56	.914
Paclitaxel + carboplatin/cisplatin	136	99	11.0	9.0-13.0	0.91	0.71-1.16	.451	0.94	0.72-1.22	.628	0.91	0.70-1.19	.506
Patients who completed four chemotherapy courses													
No	141	120	9.0	8.0-10.0	1	Reference		1	Reference		1	Reference	
Yes	498	368	12.0	10.0-13.0	0.72	0.59-0.89	.002	0.79	0.61-1.01	.063	0.78	0.61-0.99	.047
Creatinine per SD	-	-	-	-	1.09	1.00-1.18	.051	0.96	0.87-1.07	.508	0.97	0.87-1.08	.549
Serum albumin per SD	-	-	-	-	1.06	0.97-1.16	.217	1.06	0.96-1.18	.263	1.07	0.96-1.18	.228
Hemoglobin per SD	-	-	-	-	1.03	0.95-1.12	.501	1.03	0.94-1.14	.537	1.01	0.91-1.11	.867

Abbreviations: AWGS, Asian Working Group for Sarcopenia; BMI, body mass index; CT, computed tomography; ECOG PS, Eastern Cooperative Oncology Group performance status; LBM, lean body mass; SD, standard deviation; SMA, skeletal muscle cross-sectional area; SMI, skeletal muscle index (skeletal muscle area/height^2); T12, 12th thoracic vertebra.

[a]Sensitivity analysis was performed using the lowest quartile of sex-specified T12 SMI to define low skeletal muscle mass. Subsequently, CT-defined sarcopenia, AWGS-defined sarcopenia, and AWGS-defined severe sarcopenia were accordingly re-determined. Afterwards, univariate and multivariate analyses were reperformed using Cox proportional hazards models.

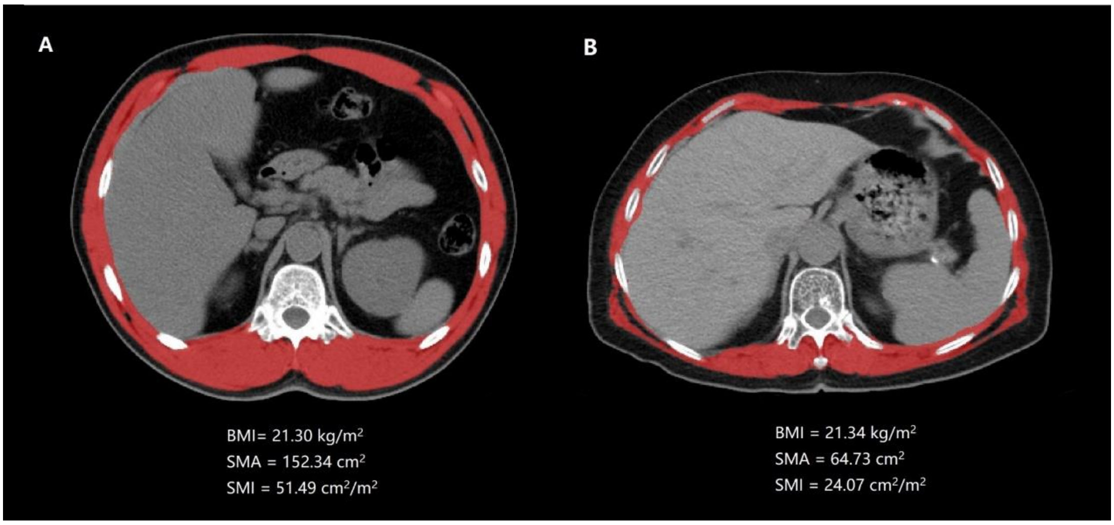

eFigure 1. Cross-Sectional Computed Tomography (CT) Images at the 12th Thoracic Vertebra Level Used for the Quantification of the Skeletal Muscle Area (T12 SMA)

(A) and (B) show the findings of two men with stage IV NSCLC. The BMIs of the two patients were almost identical. The red color indicates T12 SMA. Based on our diagnostic criteria, patient A had no CT-defined sarcopenia, whereas patient B had CT-defined sarcopenia. BMI, body mass index; SMI, skeletal muscle index.

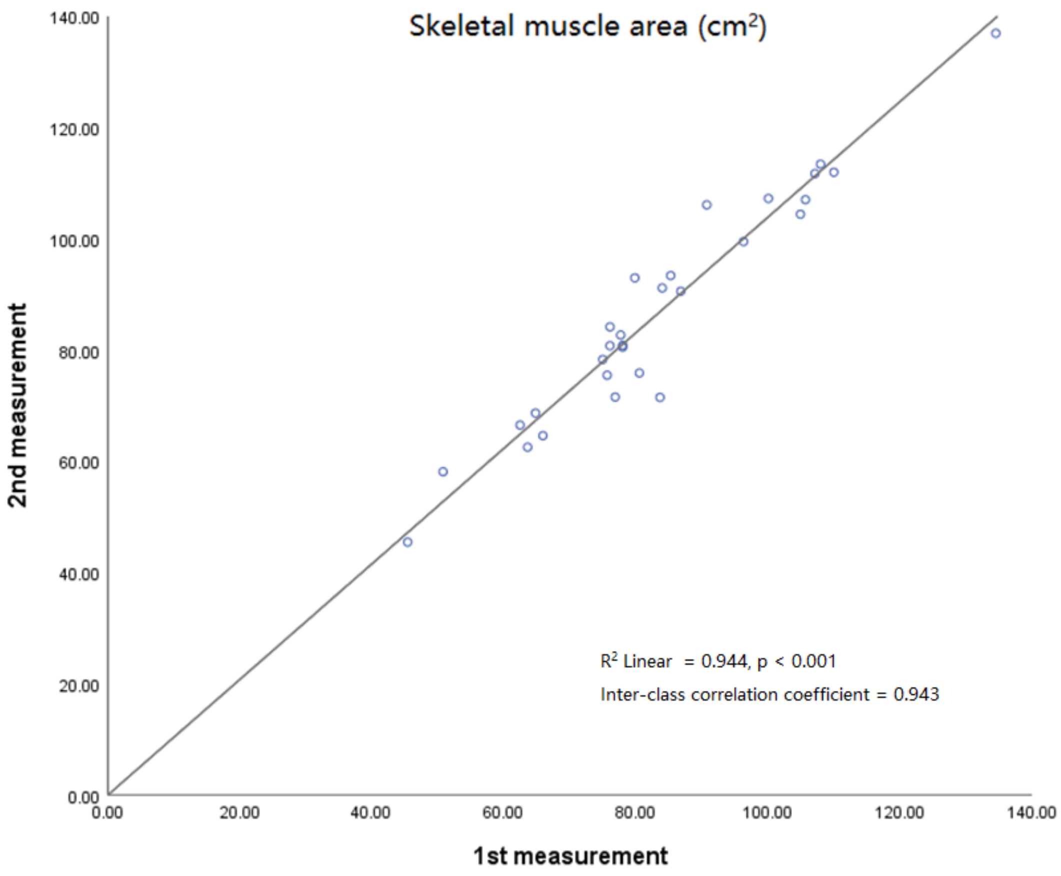

eFigure 2. Inter-observer Validation Using Interclass Correlation Coefficient Analysis

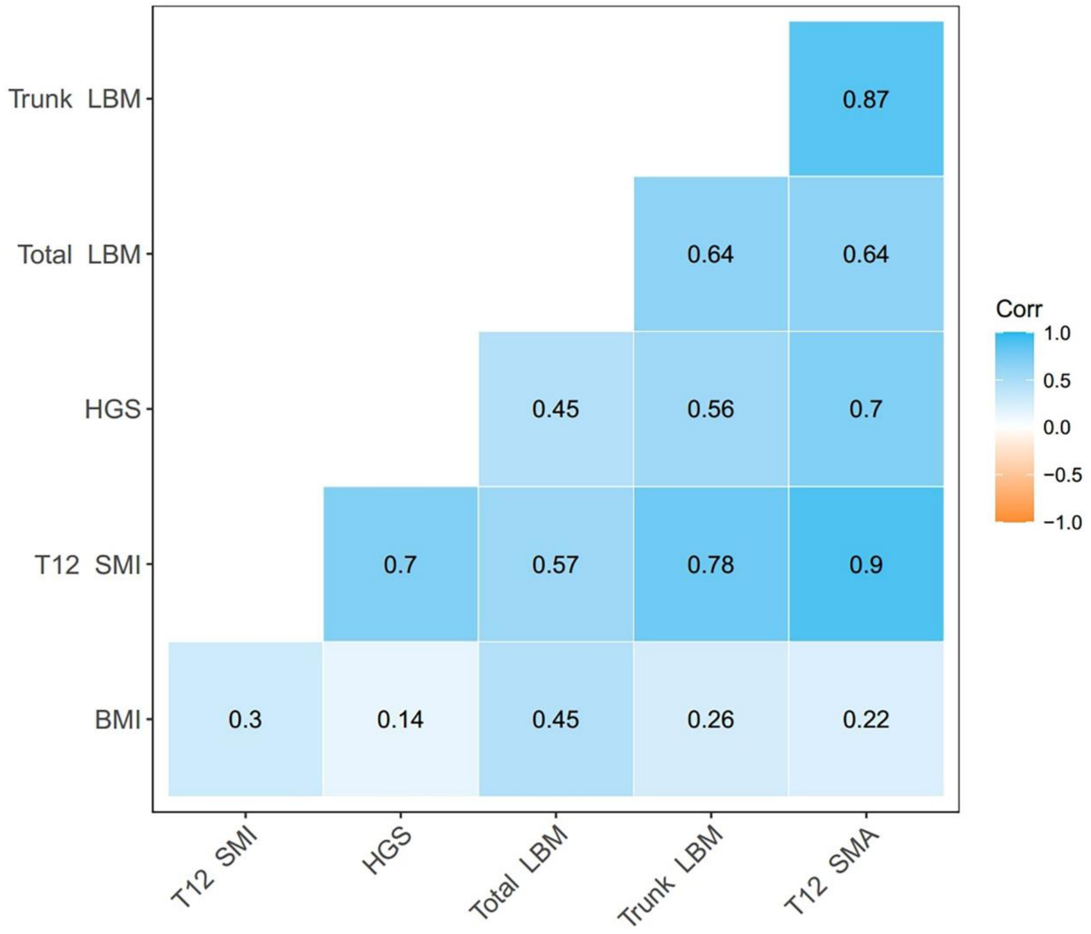

eFigure 3. Correlation Matrix of Total Lean Body Mass (LBM), Trunk LBM, Handgrip Strength (HGS), Skeletal Mass Index at the 12th Thoracic Vertebra Level (T12 SMI), Body Mass Index (BMI), and Skeletal Mass Area at the 12th Thoracic Vertebra Level (T12 SMA)

The numbers in boxes indicate Pearson's correlation coefficient (*r*).

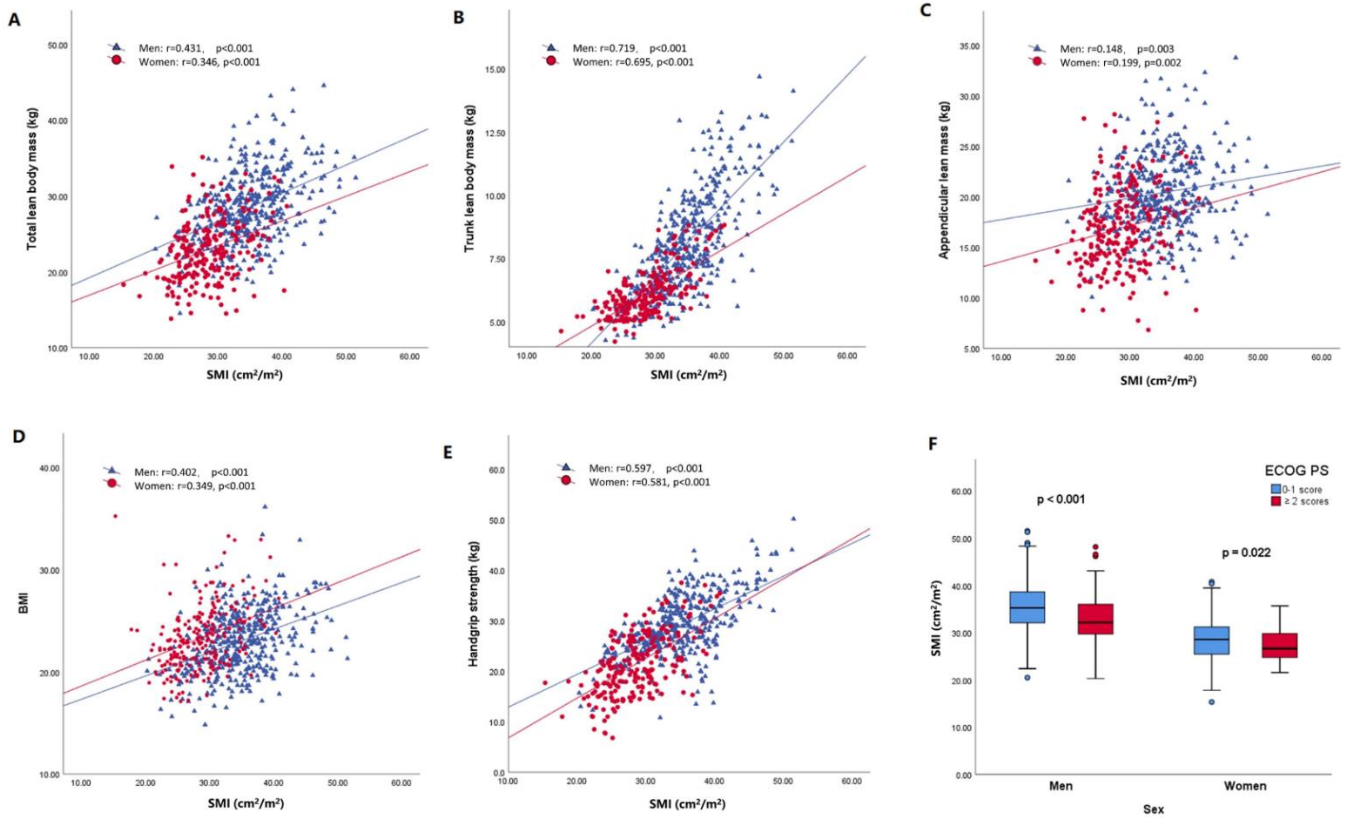

eFigure 4. Correlations of T12 SMI with (A) Total Lean Body Mass (LBM), (B) Trunk LBM, (B) Appendicular LBM, (D) BMI, and (E) Handgrip Strength

(F) illustrates the group difference in SMI between the ECOG PS = 0-1 group and the ECOG PS ≥2 group. BMI, body mass index; ECOG PS: Eastern Cooperative Oncology Group performance status; SMI, skeletal muscle index.

A

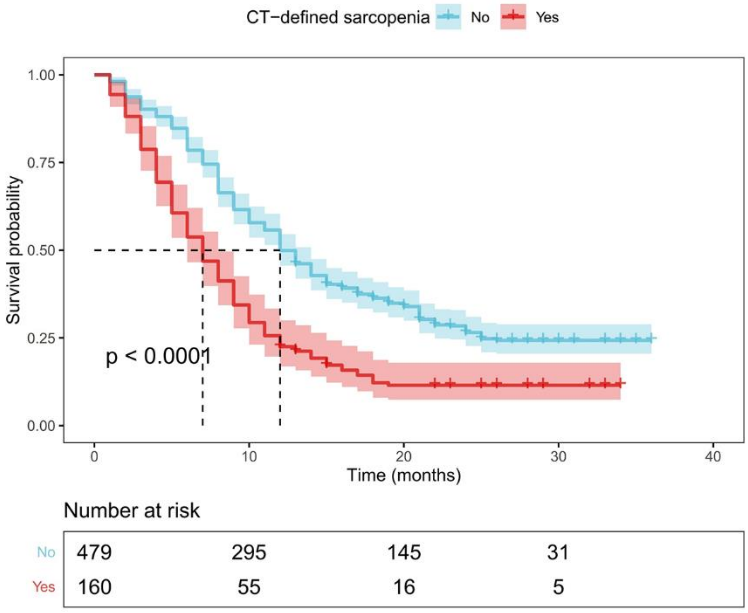

B

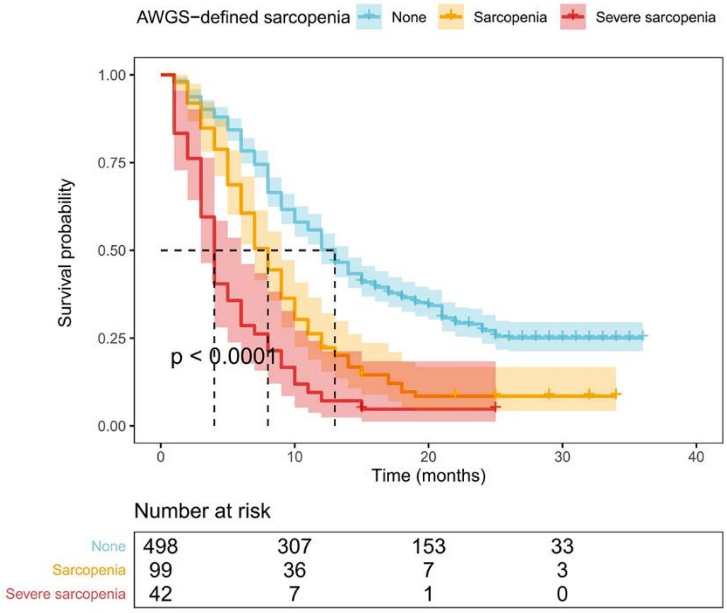

eFigure 5. Sensitivity Analysis of Kaplan–Meier Curves Showing Overall Survival in Patients with (A) CT-Defined Sarcopenia and (B) AWGS-Defined Sarcopenia or Severe Sarcopenia

Sensitivity analysis was performed using the lowest quartile of sex-specified T12 SMI to define low SMM. CT-defined sarcopenia, AWGS-defined sarcopenia, and AWGS-defined severe sarcopenia were accordingly re-determined. *P* values indicate the results of the log-rank test. Shadowed areas indicate 95% confidence intervals. AWGS, Asian Working Group for Sarcopenia; CT, computed tomography; T12 SMI, skeletal mass index at the 12th thoracic vertebra level; SMM, skeletal muscle mass.
